# Rational design of inorganic dielectric materials with expected permittivity

**DOI:** 10.1038/srep16769

**Published:** 2015-11-30

**Authors:** Congwei Xie, Artem R. Oganov, Dong Dong, Ning Liu, Duan Li, Tekalign Terfa Debela

**Affiliations:** 1 International Center for Materials Discovery, School of Materials Science and Engineering, Northwestern Polytechnical University, Xi’an, Shaanxi 710072, P.R. China; 2 Science and Technology on Thermostructural Composite Materials Laboratory, School of Materials Science and Engineering, Northwestern Polytechnical University, Xi’an, Shaanxi 710072, P.R. China; 3 Skolkovo Institute of Science and Technology, 3 Nobel St., Skolkovo 143025, Russia; 4 Moscow Institute of Physics and Technology, 9 Institutskiy Lane, Dolgoprudny City, Moscow Region 141700, Russia; 5 Department of Geosciences and Center for Materials by Design, Stony Brook University, Stony Brook, New York 11794, USA

## Abstract

Techniques for rapid design of dielectric materials with appropriate permittivity for many important technological applications are urgently needed. It is found that functional structure blocks (FSBs) are helpful in rational design of inorganic dielectrics with expected permittivity. To achieve this, coordination polyhedra are parameterized as FSBs and a simple empirical model to evaluate permittivity based on these FSB parameters is proposed. Using this model, a wide range of examples including ferroelectric, high/low permittivity materials are discussed, resulting in several candidate materials for experimental follow-up.

Dielectric materials are essential for many technological applications in optical, electronic, and micro-electronic devices. For instance, high-permittivity materials are required for gate dielectrics and high-energy storage capacitors, and low-permittivity dielectrics are necessary for transparent windows and miniaturized integrated circuits. The search for these dielectric materials over a wide range of compounds is time-consuming. A major reason is the lack of a clear and intuitive data set to give an idea about which materials should be focused on^1^. Fortunately, we now have computational tools such as codes based on density functional theory^2^,^3^ (DFT), capable of accurately predicting many important materials properties. With the help of computations, materials discovery can be accelerated^4^,^5^,^6^.

Up to now, high-throughput computational approach have been employed to screen thousands of compounds for new materials^7^,^8^,^9^,^10^,^11^,^12^,^13^,^14^. Structure prediction methods^15^, such as USPEX^16^,^17^, have also been developed to optimize certain properties of materials with only the chemical composition given^18^,^19^,^20^,^21^. However, the efficiency of these theoretical methods requires a fast and accurate evaluation of the properties of interest, while dielectric properties are relatively time-consuming. Therefore, it would be desirable to find a way to compute them from crystal structure, most transparently using functional structure blocks (FSBs), which are directly linked to the materials properties. The application of this FSB method mainly depends on: (1) the determination of a suitable FSB for a certain property of materials; and (2) the establishment of an explicit relationship between this property and its FSB. With such structure-property relations, one can quantitatively or qualitatively evaluate properties for a material in seconds. In this paper, we will demonstrate that the idea of FSBs could be very useful for rational design of materials with expected permittivity.

Inspired by Rignanese *et al.*^22^ and our previous studies^21^,^23^, we choose the coordination polyhedron as FSB for permittivity due to its major and easy to rationalize effect on permittivities of materials. Coordination polyhedron to a very large extent determines many aspects of lattice dynamics and thus can be used to determine permittivity^21^,^22^. Rignanese *et al.*^22^ proposed an empirical model to calculate permittivity, for each coordination polyhedron using three characteristic parameters (electronic polarizability 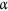, charge 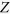, and force constant 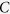). In this present study, we suggest a simplified empirical model with each type of coordination polyhedra characterized by two parameters: electronic polarizability 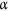 and ionic oscillator strength 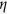. Furthermore, by introducing the volume 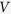 of each type of polyhedron, we can extend our model to estimate permittivity of a crystal structure provided that the type of coordination polyhedron is known. This means that dielectric materials with expected permittivity could be constructed by selecting appropriate coordination polyhedra.

## Results and Discussions

### Description of the model

According to Rignanese’s model^22^, it is possible to evaluate the electronic^24^, lattice, and static permittivities of a given structure based on its electronic polarizability 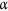, charge 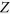, and force constant 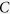:


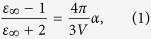



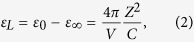


where 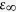 is the electronic permittivity; 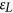 is the lattice permittivity; 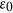 is the static permittivity; and 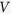 is the volume of the structure. They define 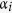, 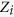, and 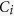 values for each type of coordination polyhedron *i*, and assuming that:


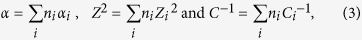


where 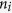 is the number of type-*i* coordination polyhedron contained in a structure. Summation is done over all types of coordination polyhedra. The optimal 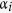, 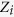, and 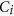 values for each type of coordination polyhedron *i* can be determined using least-squares method based on the 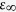, 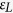, and 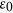 values calculated from first principles for a set of materials. However, 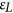 obtained by their model is sometimes very different from that calculated from first principles. This may be due to the fact that 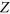 and 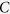 are considered as two independent variables in their model, which, however, may be correlated to each other. Therefore, we suggest defining a single parameter of ionic oscillator strength 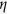:


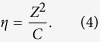


Then, the lattice permittivity 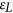 can be calculated as:


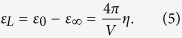


By analogy with 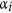, we define 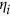 for each type of coordination polyhedron *i* such that:


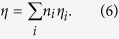


The optimal values 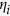 can be determined in the same way as for 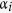. As shown in the following part of this paper, 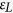 obtained from our simplified model improve upon those calculated from Rignanese’s model in most cases.

### Test of the model

We have calculated permittivity of various inorganic compounds constructed from three binary oxide systems (MgO, Al_2_O_3_, and SiO_2_). With the crystal structures of these compounds obtained from Materials Project^1^, we performed full structure relaxation before calculating permittivity using the density functional perturbation theory (DFPT^25^) approach. Structural information and DFPT permittivities of these compounds can be found as Supplementary Table Is. The optimal 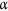 and 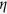 values of seven coordination polyhedra, MgO_4_, MgO_6_, AlO_4_, AlO_5_, AlO_6_, SiO_4_, and SiO_6_ obtained in our model are listed in Table 1.

**Figure 1:**
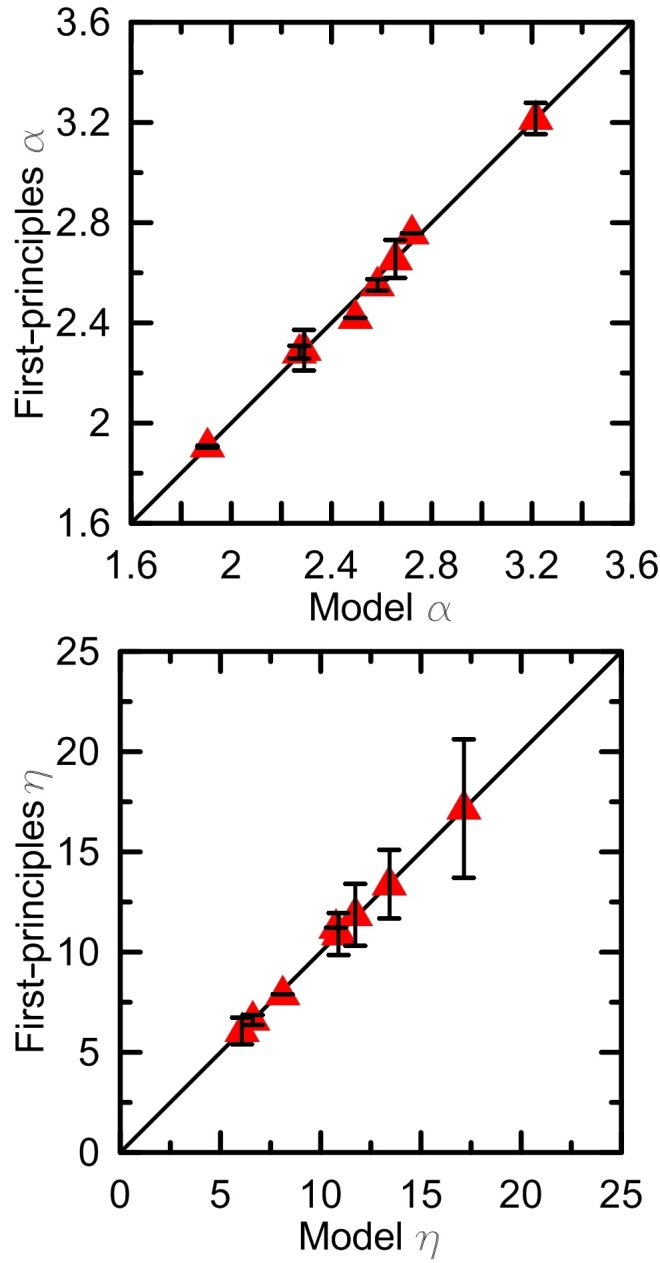
Characteristic parameters *α* and *η*. Comparison between characteristic parameters *α* (in Å^3^) and *η* (in Å^3^) of many MgO, Al_2_O_3_, and SiO_2_ phases calculated from DFPT and those derived from optimal *α*_*i*_ and *η*_*i*_ values reported for coordination polyhedron *i*.

In Fig. 1, 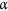 and 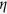 values of MgO, Al_2_O_3_, and SiO_2_ compounds given by our model are compared to those calculated from DFPT approach, with quite good agreement for most of the structures. In particular, 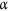 values obtained in our model agree very well with those computed by the DFPT approach, with an average relative error as low as 1.5%. Although a few 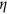 values have error higher than 10%, it can be concluded that our 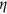 values of MgO_4_, MgO_6_, AlO_4_, AlO_5_, AlO_6_, SiO_4_, and SiO_6_ coordination polyhedra are reliable.

To test the applicability of our model, we evaluated permittivities of many ternary and quaternary oxides in (MgO)_*x*_(Al_2_O_3_)_*y*_(SiO_2_)_*z*_ system (see Table 2). DFPT results obtained by us and some experimentally or theoretically reported values are also listed in Table 2 for comparison. We can see that our model with optimized 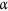 and 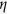 is really helpful to evaluate materials permittivity. Moreover, our model may provide a way to obtain permittivity for very complex systems where DFPT approach is not feasible, e.g., enstatite MgSiO_3_ (80 atoms/cell) listed in Table 2.

However, one must keep in mind the limitations of the model (see 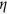 values shown in Fig. 1). We conclude that our simplified model is not suitable for materials with low-frequency polar modes having large contributions (due to large 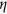 values) to the lattice permittivity. We return to this point later in this paper.

**Figure 2:**
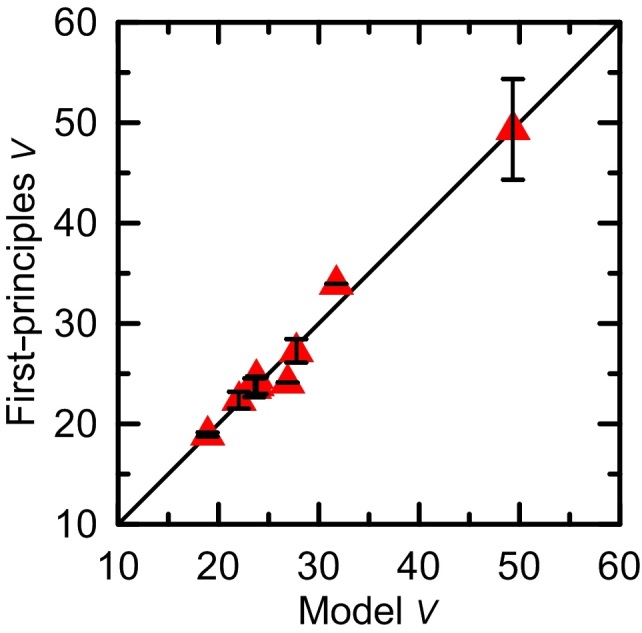
Volume *V*. Comparison between volume *V* (in Å^3^) of many MgO, Al_2_O_3_, and SiO_2_ compounds calculated from DFPT and those derived from optimal *V*_*i*_ values reported for coordination polyhedron *i*.

Our model can also be extended to evaluate permittivity of a hypothetical structure, for which only the types of coordination polyhedra are given. To achieve this, we define volume 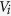 for each type of coordination polyhedron *i*, and determine optimal 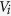 values in the same way as for 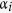 and 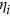 (as listed in Table 1). The addition of 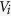 of coordination polyhedron *i* can reproduce volume of a structure well (as shown in Fig. 2). Then the 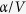 (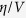) values of a structure can be obtained from:


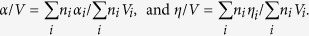


**Figure 3:**
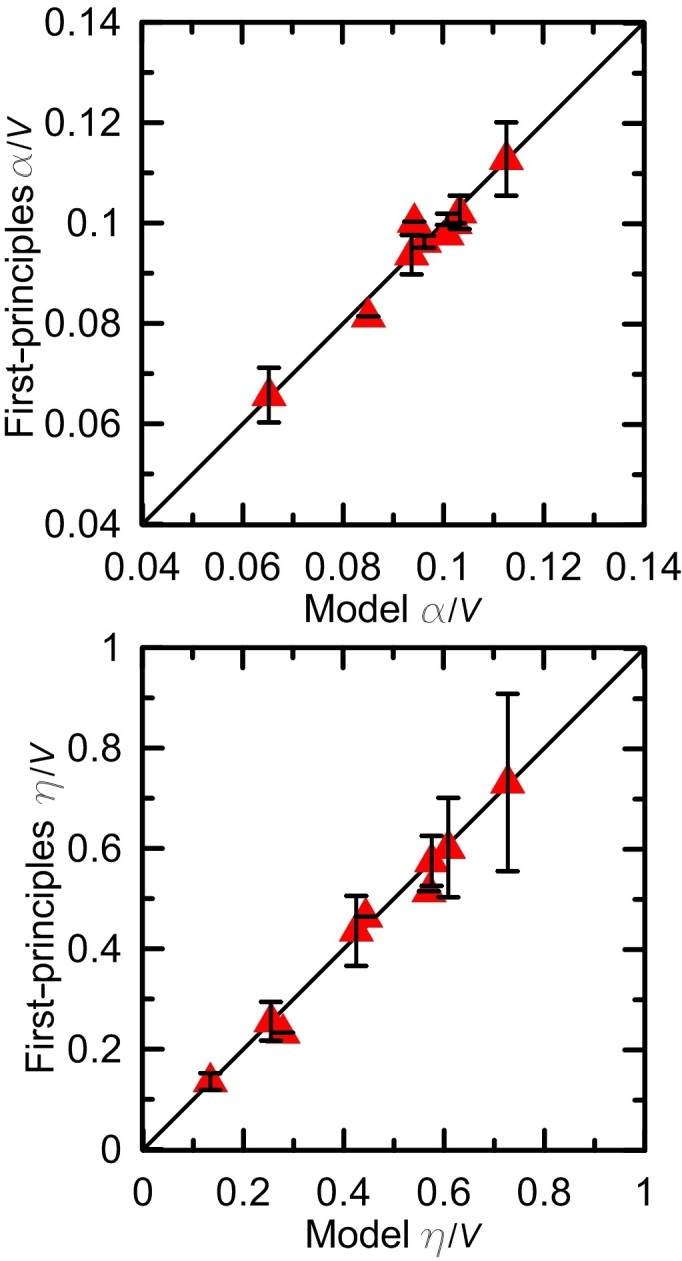
Parameters *α*/*V* and *η*/*V*. Comparison between parameters (***α/V*** and ***η*****/*****V***) of many MgO, Al_2_O_3_, and SiO_2_ phases calculated from DFPT and those estimated by using *α*_*i*_, *η*_*i*_, and *V*_*i*_ values of coordination polyhedron *i*.

The corresponding 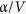 (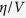) values are comparable to those calculated from DFPT approach (see Fig. 3). In this way, permittivity of a hypothetical structure can be reasonably evaluated.

### Application of the model

The 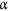, 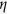, and 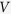 values of each type of coordination polyhedra obtained from our model are helpful to design dielectric materials with expected permittivity. First, we extended our model to study some other oxides, nitrides, and fluorides (see Supplementary Table IIs). We obtained 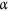, 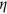, and 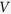 values for another 19 coordination polyhedra (see Table 1). With the 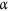, 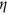, and 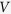 values of 26 coordination polyhedra listed in Table 1, we illustrated how to rationally design ferroelectric, and high/low permittivity materials.

**Figure 4:**
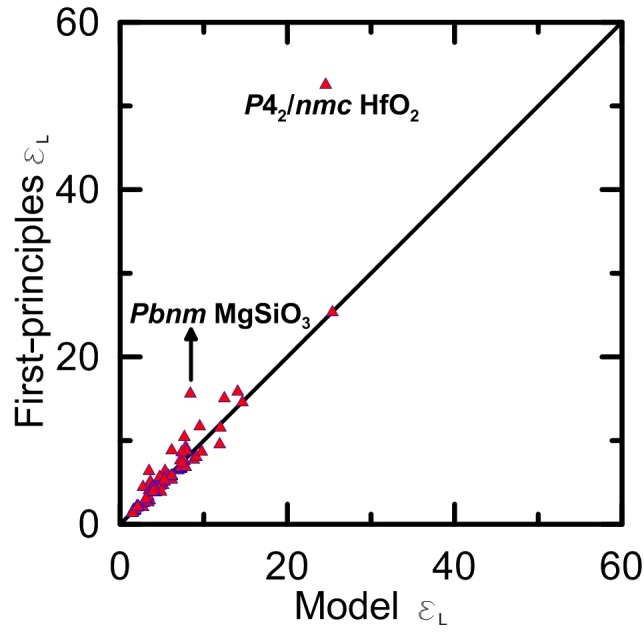
Lattice permittivity *ε*_*L*_. Comparison between lattice permittivity *ε*_*L*_ of 95 compounds obtained by using the present simplified semi-empirical model and those calculated from DFPT.

We have calculated 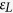 of 95 compounds using 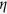 values of these 26 coordination polyhedra. Some of these compounds are listed in Table 2. The complete list of compounds can be found as Supplementary Tables Is and IIs. We compare 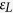 values of these 95 compounds with those calculated from DFPT approach (see Fig. 4). The agreement between the two data sets is good. However, there are two deviating structures, *P*4_2_*/nmc* HfO_2_ and *Pbnm* MgSiO_3_, for which the actual 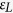 is much higher than that from our model. We found that the “unusual” enhancement of 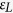 is related to large 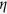 values. This may originate from low-frequency polar phonon modes, which means that these two structures can be close to a ferroelectric instability.

In fact, the *P*4_2_/*nmc* HfO_2_ is a well-known ferroelectric material. Another structure, *Pbnm* MgSiO_3_, possesses a perovskite structure adopted by many ferroelectric materials. We calculated the contributions to 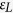 from each polar phonon mode of *Pbnm* MgSiO_3_ (as listed Table 3). The *Pbnm* MgSiO_3_ indeed possesses a low-frequency polar phonon mode (at 175 cm^−1^) contributing to 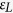 much more than other phonon modes. In other words, our model underestimates permittivities of ferroelectrics and crystals with softened polar modes. This can actually be used for rapid screening of potential ferroelectric materials.

Our model is also helpful in the design of materials with high/low permittivity. Our results show, quite intuitively, that coordination polyhedra with high 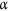 (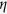), and low 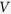 are favorable for high dielectric permittivity.

At a glance at Table I, we can find that HfO_8_ has much higher 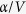 and 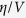 values than others among the 26 coordination polyhedra. Indeed, Hf oxides are excellent high-permittivity oxides (ref. 20). On the other hand, SiO_4_ tetrahedron possesses the lowest 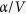 and 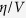 values among O-based coordination polyhedra. Indeed, SiO_2_ (quartz and silica glass) with SiO_4_ tetrahedra is a well-known low-permittivity material in micro-electronics industry.

Noticeably, 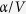 and 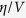 values of N-based coordination polyhedra are higher than those of O-based coordination polyhedra. For instance, AlN_6_ coordination polyhedron has much higher 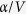 and 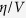 values than AlO_6._ We may expect high-permittivity in nitrides, e.g., Hf_3_N_4_ with HfN_8_ coordination polyhedron. As listed in Table 1, 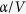 and 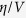
values of HfN_8_ coordination polyhedron are higher than those of the HfO_8_ polyhedron. Therefore, 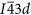 Hf_3_N_4_ with HfN_8_ coordination polyhedron has higher permittivities than most of hafnium oxides (see Supplementary Table IIs).

**Figure 5:**
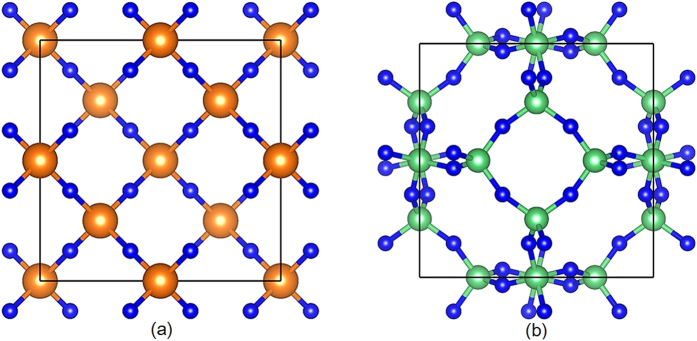
Crystal structures of MgF_2_ and BeF_2_. (**a**) 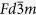 MgF_2_ constructed from MgF_4_ coordination polyhedra; (**b**) 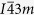 BeF_2_ constructed from BeF_4_ coordination polyhedra. Blue spheres denote F atoms, brown spheres denote Mg atoms, and green spheres denote Be atoms.

For the design of low-permittivity materials, we can immediately expect that permittivity of an oxide can be decreased by replacing O with F (see Table 1). Experimentally, SiF_4_ material with SiF_4_ tetrahedra has much lower permittivity than quartz^26^,^27^. In a similar way, we can expect that 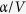 and 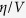 values of MgF_4_ coordination polyhedron may be much lower than those of MgO_4_ polyhedron. Therefore, we try to design low-permittivity MgF_2_ material with MgF_4_ coordination polyhedron. We constructed a new 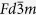 MgF_2_ phase (Fig. 5(a)) with very low permittivity using 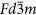 SiO_2_ structure (cristobalite) with SiO_4_ tetrahedra (detailed structural information can be found as Supplementary Table IIIs). The static permittivity 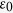 of 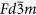 MgF_2_ (2.5) is much lower than that of quartz (3.9^27^) and comparable to most low-permittivity polymers. The dynamical and mechanical stability of 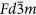 MgF_2_ was verified by phonon and elastic constants calculations (see Supplementary Fig. 1s and Table IVs). The enthalpy of 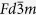 MgF_2_ phase is only 0.1 eV/atom higher than that of the most stable MgF_2_ structure (*P*4_2_*/mnm* phase). Moreover, this inorganic material may have a better mechanical strength than polymers (see Supplementary Table IVs). This suggests that 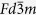 MgF_2_ may be synthesized and tested as a potential low-permittivity material.

From the Materials Project, we also found a near-ground-state BeF_2_ structure (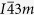) with BeF_4_ coordination polyhedra, as shown in Fig. 5(b). The static permittivity 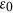 of 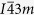 BeF_2_ is 2.5, indicating that BeF_2_ is also a good low-permittivity material. We suggest that compounds constructed from LiF_4_, BF_4_, NaF_4_, and AlF_4_ coordination polyhedra may also have low permittivities, e.g., 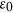 of *P*3_1_21 LiBF_4_ with LiF_4_ and BF_4_ coordination polyhedra can be as low as 3.6.

We have to mention that coordination number is an important factor to design high/low-permittivity materials. There is a trend^21^,^23^: low coordination number, low permittivity. Our present study agrees with this trend well; coordination polyhedra with low coordination number have low 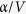 and 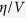 values. For example, our study shows that the 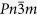 SiC_2_N_4_ structure, with 1/3 SiN_4_ and 2/3 CN_2_ coordination polyhedra, has much lower permittivity (4.6) than *P*6_3_/*m* Si_3_N_4_ (8.3) containing SiN_4_ coordination polyhedra.

To summarize, we have presented a method for designing new inorganic dielectrics with expected permittivity is discussed. Coordination polyhedron is adopted as the functional structural block (FSB) of permittivity. Three parameters (electronic polarizability 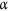, ionic oscillator strength 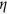, and volume 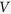) are chosen to characterize each coordination polyhedron. We show applications of this model evaluate materials permittivity. Results derived from this model agree well with those from density-functional perturbation theory. Moreover, 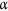, 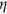, and 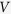 values assigned to coordination polyhedra may be helpful to make intuitive choices of materials to focus on. Successful applications include ferroelectric, high- and low-permittivity materials.

## Methods

Before calculating the properties, we perform full structure relaxation using density functional theory (DFT^2^,^3^) as implemented in the Vienna *ab intio* Simulation Package (VASP^28^) with the PBEsol-GGA^29^,^30^ exchange-correlation functional. The all-electron projector-augmented wave (PAW) method^31^ is used, with a plane-wave energy cutoff of 900 eV and *k*-point meshes with reciprocal-space resolution of 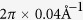. These settings enable excellent convergence for the energy differences, stress tensors, and structural parameters. With fully relaxed structures, dielectric^25^ and mechanical^32^ properties (e.g. the elastic constants) were computed. Permittivities and phonon dispersion curves are calculated using density functional perturbation theory (DFPT^25^). Phonon dispersion curves were obtained by PHONOPY^33^.

## Additional Information

**How to cite this article**: Xie, C. *et al.* Rational design of inorganic dielectric materials with expected permittivity. *Sci. Rep.* **5**, 16769; doi: 10.1038/srep16769 (2015).

## Supplementary Material

Supplementary Information

## Figures and Tables

**Table 1:** Electronic polarizabilities ( 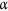 in Å^3^), ionic oscillator strengths ( 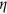 in Å^3^), effective volumes ( 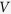 in Å^3^), electronic polarizabilities per volume ( 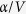 ), and ionic oscillator strengths per volume ( 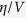 ) of 26 coordination polyhedra.

Coordinationpolyhedron	*α*	*η*	*V*	*α*/*V*	*η*/*V*
LiO_4_	1.16	4.79	12.42	0.093	0.386
LiF_6_	1.03	11.54	16.75	0.061	0.689
BeO_4_	1.39	4.54	14.03	0.099	0.323
BeF_4_	1.83	4.53	44.99	0.041	0.101
BO_3_	2.17	4.25	24.47	0.089	0.173
BO_4_	1.84	5.09	19.16	0.096	0.266
NaO_4_	2.3	7.22	21.21	0.108	0.341
NaF_6_	1.24	7.16	24.66	0.050	0.291
MgN_4_	3.08	8.76	20.81	0.148	0.421
MgO_4_	2.29	6.07	23.82	0.096	0.255
MgO_6_	1.91	10.89	18.92	0.101	0.575
MgF_6_	2.00	9.06	33.58	0.060	0.270
AlN_4_	2.77	6.94	21.30	0.130	0.326
AlN_6_	2.34	19.57	16.85	0.139	1.161
AlO_4_	2.72	8.10	31.71	0.086	0.255
AlO_5_	2.45	15.35	23.91	0.102	0.642
AlO_6_	2.27	13.44	22.06	0.103	0.609
AlF_6_	2.69	11.21	47.17	0.057	0.238
SiN_4_	3.13	7.71	24.86	0.126	0.310
SiN_6_	2.56	12.45	17.15	0.149	0.726
SiO_4_	3.21	6.61	49.33	0.065	0.134
SiO_6_	2.66	17.15	23.61	0.112	0.726
HfO_6_	5.17	31.84	32.22	0.120	0.737
HfO_7_	4.61	40.24	34.48	0.134	1.167
HfO_8_	4.49	53.40	32.36	0.139	1.650
HfN_8_	4.63	52.39	24.99	0.185	2.096

**Table 2:** Space group (SG), and permittivities (electronic − 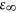 , and static− 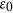 ) of some ternary and quaternary oxides in the (MgO)_*x*_(Al_2_O_3_)_*y*_(SiO_2_)_*z*_ system.

Compound	SG	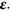			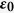		
		model	DFPT	reported	model	DFPT	reported
MgAl_2_O_4_ (Spinel)	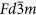	3.18	3.06	2.89^34^	9.27	8.51	8.40^35^,8.75^36^
MgAl_2_O_4_ (CaFe_2_O_4_-type)	*Pbnm*	3.46	3.31		11.36	15.13	
MgAl_2_O_4_ (CaTi_2_O_4_-type)	*Cmcm*	3.36	3.30		11.07	14.46	
MgSiO_3_ (Enstatite)	*Pbca*	3.11	–		7.35	–	8.23^37^
							
MgSiO_3_ (Clinoenstatite)	*P*2_1_*/c*	3.09	2.82		7.30	9.25	
							
MgSiO_3_ (Protoenstatite)	*Pnab*	2.88	2.78		6.84	7.10	6.70^38^
							
MgSiO_3_ (Clinoenstatite)	*C*2*/c*	2.88	2.78		6.83	7.31	
							
MgSiO_3_ (Corundum)	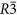	3.20	3.15		11.00	10.07	
MgSiO_3_ (Perovskite)	*Pbnm*	3.52	3.38		11.94	16.80	
Mg_2_SiO_4_ (Forsterite)	*Pbnm*	2.96	2.84	2.78^39^	7.76	7.52	6.80^40^,7.30^41^
Mg_2_SiO_4_ (Wadsleyite)	*Imma*	3.21	3.01		8.39	8.45	
Mg_2_SiO_4_ (Ringwoodite)	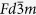	3.33	3.03		8.64	8.14	
Al_2_SiO_5_ (Andalusite)	*Pmnn*	2.78	2.83	2.78^42^	7.51	7.79	8.28^37^,8.0^43^
Al_2_SiO_5_(Sillimanite)	*Pmcn*	2.97	2.88	2.85^42^	7.16	7.47	9.29^37^,6.2^44^
Al_2_SiO_5_ (Kyanite)	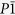	3.24	3.09	3.14^42^	8.78	8.78	
Mg_2_Al_4_Si_5_O_18_(Cordierite)	*Cccm*	2.42	2.39		5.34	4.97	5.0^45^,6.14^46^

**Table 3:** Frequencies of polar phonon modes ( 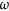 [cm^−1^]) and their contributions to the permittivity ( 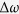 ) computed for *Pbnm* MgSiO_3_ 46.

Mode	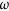 [cm^−1^]	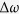	Mode	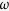 [cm^−1^]	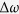	Mode	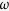 [cm^−1^]	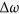
B_2u_	175	6.14	B_2u_	430	1.83	B_3u_	662	0.11
B_3u_	239	0.61	B_1u_	449	0.14	B_1u_	688	~0
B_1u_	253	0.24	B_2u_	464	0.64	B_2u_	690	0.02
B_2u_	293	0.83	B_3u_	474	2.02	B_2u_	715	0.20
B_1u_	307	1.80	B_1u_	486	1.80	B_3u_	737	0.22
B_3u_	332	1.04	B_3u_	514	0.07	B_3u_	749	~0
B_3u_	367	0.23	B_1u_	541	~0	B_1u_	760	0.16
B_3u_	405	0.63	B_1u_	582	0.37			
B_1u_	416	0.30	B_2u_	586	0.23			
