# Supplementary material for: Rational design of inorganic dielectric materials with expected permittivity
Source: Sci Rep. 2015 Nov 30;5:16769. doi: 10.1038/srep16769 (PMC4663754; doi:10.1038/srep16769)
Supplement: Supplementary Information [file srep16769-s1.pdf]

Rational design of inorganic dielectric materials with expected permittivity

Congwei Xie [a, b, *], Artem R. Oganov [c, d, a, e †], Dong Dong [a, b], Ning Liu [a, b], Duan Li [a, b], Tekalign Terfa Debela [a, b]

[a]*International Center for Materials Discovery, School of Materials Science and Engineering, Northwestern Polytechnical University, Xi'an, Shaanxi 710072, PR China*
[b]*Science and Technology on Thermostructural Composite Materials Laboratory, School of Materials Science and Engineering, Northwestern Polytechnical University, Xi'an, Shaanxi 710072, PR China*
[c]*Skolkovo Institute of Science and Technology,5 Nobel street, Skolkovo 143025, Russia*
[d]*Moscow Institute of Physics and Technology, 9 Institutskiy Lane, Dolgoprudny City, Moscow Region 141700, Russia*
[e]*Department of Geosciences and Center for Materials by Design, Stony Brook University, Stony Brook, New York 11794, USA*

* xiecw1021@mail.nwpu.edu.cn
† artem.oganov@stonybrook.edu

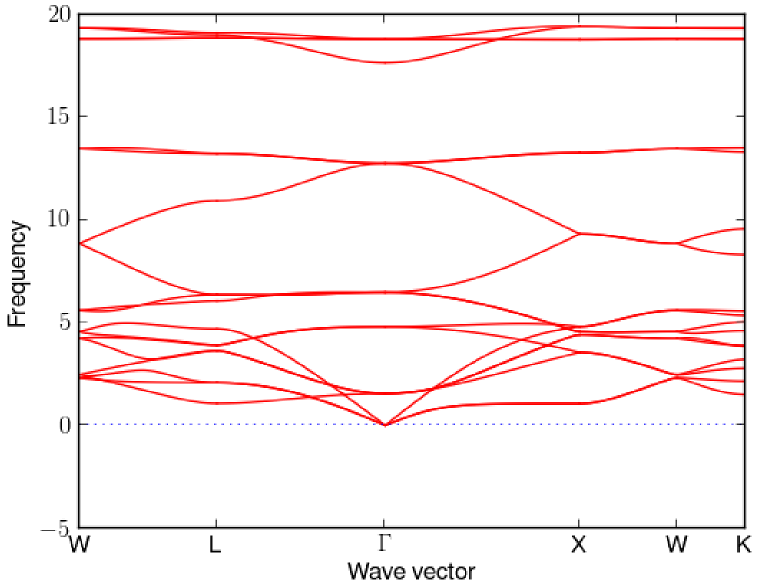

Figure 1s Phonon dispersion curves of *Fd*-3*m* MgF_2.

Table Is Structural information (space group SG, materials project number MP-Id, coordination polyhedron type CP-type, and average volume per coordination polyhedron *V*), permittivities (electronic ε_∞, lattice ε_L, and static ε_0), and characteristic parameters (average electronic polarizability per coordination polyhedron α and average ionic oscillator strength per coordination polyhedron η) of some MgO, Al_2O_3, and SiO_2 compounds.

MgO polymorphs								
SG	MP-Id	CP-type	V	ε_∞	ε_L	ε_0	α	η
*P*3*m*1	mp-775808	100% MgO_4	24.28	3.00	3.02	6.02	2.32	5.84
*P*6$_3$*mc*	mp-776911	100% MgO_4	24.18	2.98	3.10	6.08	2.30	5.96
*P*6$_3$*mc*	mp-549706	100% MgO_4	24.31	3.01	2.99	6.00	2.33	5.78
F-43*m*	mp-1778	100% MgO_4	24.06	3.09	2.89	5.98	2.36	5.53
*P*6$_3$*mmc*	not in	100% MgO_4	22.29	3.04	4.07	7.11	2.15	7.22
*I*4/*mmm*	not in	100% MgO_6	18.80	3.22	6.78	10.00	1.91	10.14
Fm-3*m*	mp-1265	100% MgO_6	19.05	3.16	7.67	10.83	1.90	11.63
Al_2O_3 polymorphs								
SG	MP-Id	CP-type	V	ε_∞	ε_L	ε_0	α	η
*Ccm*2$_1$	mp-754401	100% AlO_4	33.92	2.55	2.92	5.47	2.76	7.88
*C*2/*m*	mp-754812	50% AlO_4+50% AlO_5	28.36	2.80	5.28	8.08	2.54	11.92
Pm	not in	50% AlO_4+50% AlO_5	26.09	3.06	6.43	9.49	2.54	13.35
*P*2$_1$/*c*	mp-754531	50% AlO_4+50% AlO_5	27.32	2.96	4.72	7.68	2.58	10.26
*C*2/*m*	mp-7048	50% AlO_4+50% AlO_6	24.14	3.17	5.83	9.00	2.42	11.20
*P*2$_1$/*c*	mp-755066	100% AlO_5	24.57	3.16	6.46	9.62	2.46	12.63
P-31*c*	mp-755175	100% AlO_6	23.09	3.15	6.64	9.79	2.30	12.20
Pbcn	mp-1938	100% AlO_6	21.42	3.42	9.31	12.73	2.28	15.87
R-3	mp-754624	100% AlO_6	23.03	3.15	6.70	9.85	2.30	12.28
R-3*c*	mp-1143	100% AlO_6	21.89	3.26	7.56	10.82	2.25	13.17
SiO_2 polymorphs								
SG	MP-Id	CP-type	V	ε_∞	ε_L	ε_0	α	η
*C*2*cm*	mp-554573	100% SiO_4	47.39	2.18	1.81	3.99	3.19	6.83
*C*2/*c*	mp-12787	100% SiO_4	41.85	2.35	2.00	4.35	3.10	6.66
*C*222$_1$	mp-7648	100% SiO_4	47.68	2.18	1.76	3.94	3.21	6.68
I-42*d*	mp-546794	100% SiO_4	47.24	2.19	1.70	3.89	3.20	6.39
Cmca	mp-559313	100% SiO_4	53.24	2.04	1.62	3.66	3.27	6.86
Cmcm	mp-560527	100% SiO_4	57.33	1.95	1.41	3.36	3.29	6.43
*Ima*2	mp-555251	100% SiO_4	55.72	1.98	1.39	3.37	3.28	6.16
*P*2$_1$	mp-555891	100% SiO_4	50.06	2.11	1.59	3.70	3.23	6.33
Imma	mp-600000	100% SiO_4	57.22	1.95	1.40	3.35	3.29	6.37
I-4	mp-554151	100% SiO_4	47.66	2.18	1.76	3.94	3.21	6.67
*P*6$_3$/*mmc*	mp-7087	100% SiO_4	51.91	2.08	1.75	3.83	3.28	7.23
*P*2$_1$3	mp-8059	100% SiO_4	47.73	2.17	1.76	3.93	3.20	6.68
*P*3$_1$21	mp-7000	100% SiO_4	40.48	2.41	2.16	4.57	3.09	6.96
*P*6$_3$22	mp-559091	100% SiO_4	49.01	2.14	1.70	3.84	3.22	6.63
*P*6$_5$22	mp-554243	100% SiO_4	55.26	2.00	1.57	3.57	3.30	6.90
*P*4$_1$2$_1$2	mp-6945	100% SiO_4	46.61	2.20	1.78	3.98	3.18	6.60
Pmma	mp-639463	100% SiO_4	54.31	2.01	1.53	3.54	3.27	6.61
R-3	mp-559550	100% SiO_4	57.48	1.95	1.36	3.31	3.30	6.22
Pbcn	mp-10948	100% SiO_6	23.68	3.54	8.10	11.64	2.59	15.26
*C*2/*c*	mp-558733	100% SiO_6	24.49	3.46	7.72	11.18	2.63	15.04
*Pa*3	mp-9258	100% SiO_6	22.61	4.09	11.75	15.84	2.74	21.14

Table IIs Structural information (space group SG, materials project number MP-Id, coordination polyhedron type CP-type, and average volume per coordination polyhedron *V*), permittivities (electronic ε_∞, lattice ε_L, and static ε_0), and characteristic parameters (average electronic polarizability per coordination polyhedron α and average ionic oscillator strength per coordination polyhedron η) of some oxides, nitrides, and fluorides.

Li_2O polymorphs								
SG	MP-Id	CP-type	*V*	ε_∞	ε_L	ε_0	α	η
Fm-3*m*	mp-1960	100% LiO_4	12.42	2.92	4.85	7.77	1.16	4.79
BeO polymorphs								
SG	MP-Id	CP-type	*V*	ε_∞	ε_L	ε_0	α	η
$P6_3mc$	mp-2542	100% BeO_4	14.04	3.12	4.08	7.20	1.39	4.56
F-43*m*	mp-1778	100% BeO_4	14.01	3.14	3.97	7.13	1.39	4.43
$P4_2/mnm$	mp-7599	100% BeO_4	14.58	3.04	3.94	6.98	1.41	4.57
B_2O_3 polymorphs								
SG	MP-Id	CP-type	*V*	ε_∞	ε_L	ε_0	α	η
$P3_121$	mp-306	100% BO_3	24.47	2.77	2.18	4.95	2.17	4.25
$Cmc2_1$	mp-717	100% BO_4	19.16	3.02	3.34	6.36	1.84	5.09
Na_2O polymorphs								
SG	MP-Id	CP-type	*V*	ε_∞	ε_L	ε_0	α	η
Fm-3*m*	mp-2352	100% NaO_4	21.21	3.49	4.28	7.77	2.30	7.22
HfO_2 polymorphs								
SG	MP-Id	CP-type	*V*	ε_∞	ε_L	ε_0	α	η
C2/m	mp-755769	100% HfO_6	43.98	3.95	8.85	12.80	5.21	32.16
$I4_1/amd$	mp-754403	100% HfO_6	42.45	4.08	9.33	13.41	5.13	31.52
$P4_2/mnm$	mp-776532	100% HfO_6	38.06	4.67	23.62	28.29	5.00	71.57
Pbcn	mp-776097	100% HfO_6	37.82	4.57	21.44	26.01	4.91	64.52
$P2_1/c$	mp-352	100% HfO_7	34.90	4.77	13.35	18.12	4.64	37.07
Pbca	mp-775757	100% HfO_7	34.92	4.75	13.50	18.25	4.63	37.51
$Pc2_1b$	mp-685097	100% HfO_7	33.62	4.96	17.25	22.21	4.57	46.15
Fm-3*m*	mp-550893	100% HfO_8	32.45	5.36	32.80	38.16	4.59	84.71
P4/n	mp-775910	100% HfO_8	32.36	5.17	20.97	26.14	4.49	54.00
Pmnb	mp-741	100% HfO_8	29.88	5.59	21.92	26.51	4.31	52.12
$P4_2/nmc$	Ref 20	100% HfO_8	32.50	5.30	52.22	57.52	4.57	135.05
$MgAl_2O_4$ polymorphs								
SG	MP-Id	CP-type	*V*	ε_∞	ε_L	ε_0	α	η
Cc	Ref 21	1/3 MgO_4+2/3AlO_4	28.90	2.73	3.23	5.96	2.52	7.43
P-42*m*	Ref 21	1/3 MgO_4+2/3AlO_4	27.69	2.86	2.87	5.73	2.53	6.32
Pc	Ref 21	1/3 MgO_4+2/3AlO_4	28.85	2.73	2.62	5.35	2.52	6.02
P-4*m*2	Ref 21	1/3 MgO_4+2/3AlO_5	27.97	2.83	3.81	6.64	2.53	8.48
$P6_3/m$	Ref 21	1/3 MgO_4+2/3AlO_6	20.94	3.45	7.39	10.84	2.45	12.31
Mg_3N_2 polymorphs								
SG	MP-Id	CP-type	*V*	ε_∞	ε_L	ε_0	α	η
*Ia*3	mp-1559	100% MgN_4	20.81	5.90	5.29	11.19	3.08	8.76
AlN polymorphs								
SG	MP-Id	CP-type	*V*	ε_∞	ε_L	ε_0	α	η

F-43*m*	mp-1700	100% AlN_4	21.33	4.61	3.99	8.60	2.78	6.77
*P*6$_3$*mc*	mp-661	100% AlN_4	21.28	4.54	4.21	8.75	2.75	7.13
Fm-3*m*	mp-1330	100% AlN_6	16.85	5.18	14.59	19.77	2.34	19.57
Si_3N_4 polymorphs								
SG	MP-Id	CP-type	V	ε_∞	ε_L	ε_0	α	η
*P*6$_3$*/m*	mp-988	100% SiN_4	24.80	4.32	3.90	8.22	3.11	7.70
*P*31*c*	mp-2245	100% SiN_4	24.93	4.34	3.89	8.23	3.14	7.72
Fd-3*m*	mp-2075	1/3SiN_4+2/3SiN_6	19.69	5.35	7.40	12.75	2.78	11.59
Hf_3N_4 polymorphs								
SG	MP-Id	CP-type	V	ε_∞	ε_L	ε_0	α	η
I-43*d*	mp-11660	100% HfN_8	24.99	11.42	26.34	37.76	4.63	32.16
LiF polymorphs								
SG	MP-Id	CP-type	V	ε_∞	ε_L	ε_0	α	η
*P*6$_3$*mc*	mp-776911	100% LiF_4	20.68	1.86	2.77	4.63	1.10	4.56
Fm-3*m*	mp-1138	100% LiF_6	16.75	2.04	8.66	10.70	1.03	11.54
BeF_2 polymorphs								
SG	MP-Id	CP-type	V	ε_∞	ε_L	ε_0	α	η
I-43*m*	mp-561543	100% BeF_4	52.14	1.52	1.01	2.53	1.84	4.23
*P*6$_2$22	mp-558118	100% BeF_4	37.84	1.59	1.60	3.19	1.81	4.82
NaF polymorphs								
SG	MP-Id	CP-type	V	ε_∞	ε_L	ε_0	α	η
*P*6$_3$*mc*	mp-776911	100% NaF_4	29.79	1.68	2.25	3.93	1.31	5.33
Fm-3*m*	mp-682	100% NaF_6	24.66	1.80	3.65	5.45	1.24	7.16
MgF_2 polymorphs								
SG	MP-Id	CP-type	V	ε_∞	ε_L	ε_0	α	η
Fd-3m	not in	100% MgF_4	80.83	1.41	1.04	2.55	2.32	6.69
*P*4$_2$*/mnm*	mp-1249	100% MgF_6	33.58	2.01	3.39	5.40	2.00	9.06
AlF_3 polymorphs								
SG	MP-Id	CP-type	V	ε_∞	ε_L	ε_0	α	η
Pm-3*m*	mp-8039	100% AlF_6	48.13	1.92	2.97	4.89	2.70	11.38
R-3*c*	mp-468	100% AlF_6	46.20	1.96	3.01	4.97	2.67	11.07

Table IIIs Geometry information of *Fd*-3*m* MgF_2.

Compound	Space group	Lattice constants	Atom (Wyckoff lett.), fractional postion.			
			atom	*x*	*y*	*z*
MgF_2	*Fd*-3*m*	*a*=5.01	Mg(8a)	0.250	0.750	0.750
			F (16c)	0.375	0.875	0.625

Table IVs Elastic constants C_{ij} (GPa), bulk modulus B (GPa), shear modulus G (GPa), and Young's modulus E (GPa) computed for MgF_2 at the ground state.

Compound	C_{11}	C_{12}	C_{44}	B	G	E
MgF_2	41.9	35.6	11.1	37.7	6.7	19.0
